# The Italian version of the Physical Therapy Patient Satisfaction Questionnaire - [PTPSQ-I(15)]: psychometric properties in a sample of inpatients

**DOI:** 10.1186/1471-2474-15-135

**Published:** 2014-04-23

**Authors:** Carla Vanti, Paolo Pillastrini, Marco Monticone, Daniele Ceron, Francesca Bonetti, Raffaella Piccarreta, Andrew Guccione, Francesco Saverio Violante

**Affiliations:** 1 Department of Biomedical and Neuromotor Sciences, Alma Mater Studiorum University of Bologna, Bologna, Italy; 2 Operative Unit of Physical and Rehabilitation Medicine, Scientific Institute of Lissone, Salvatore Maugeri Foundation, IRCCS, Milan, Italy; 3 Clinical Tutor Master in Manual Therapy and Musculoskeletal Rehabilitation, University of Padova, Padova, Italy; 4 Physioup Bonetti Physiotherapy Studio, Rome, Italy; 5 Department of Decision Sciences, L.Bocconi University, Milano, Italy; 6 Department of Rehabilitation Science, College of Health and Human Services, George Mason University, Fairfax, VA 22030, USA; 7 Department of Medical and Surgical Sciences, Alma Mater Studiorum University of Bologna, Bologna, Italy

**Keywords:** Patient satisfaction, Physical therapy, Hospital, Quality of health care, Outcome assessment (health care)

## Abstract

**Background:**

In a previous study we described the translation, cultural adaptation, and validation of the Italian version of the PTPSQ [PTPSQ-I(15)] in outpatients. To the authors’ knowledge, the PTPSQ was never studied in a hospital setting.

The aims of this study were: (1) to establish the psychometric properties of the Physical Therapy Patient Satisfaction Questionnaire [PTPSQ- I(15)] in a sample of Italian inpatients, and (2) to investigate the relationships between the characteristics of patients and physical therapists and the indicators of satisfaction.

**Methods:**

The PTPSQ-I(15) was administered to inpatients in a Physical Medicine and Rehabilitation Unit. Reliability of the PTPSQ-I(15) was measured by internal consistency (Cronbach’s α) and test-retest stability (ICC 3,1). The internal structure was investigated by factor analysis. Divergent validity was measured by comparing the PTPSQ-I(15) with a Visual Analogue Scale (VAS) for pain and with a 5-point Likert-type scale evaluating the Global Perceived Effect (GPE) of the physical therapy treatment.

**Results:**

The PTPSQ-I(15) was administered to 148 inpatients, and 73 completed a second administration. The PTPSQ-I(15) showed high internal consistency (α = 0.949) and test-retest stability (ICC = 0.996). Divergent validity was moderate for the GPE (r = − 0.502, *P* < 0.001) and strong for the VAS (r = −0.17, *P* = 0.07). Factor analysis showed a one-factor structure.

**Conclusions:**

The administration of PTPSQ-I(15) to inpatients demonstrated strong psychometric properties and its use can be recommended with Italian-speaking population. Further studies are suggested on the concurrent validity and on the psychometric properties of the PTPSQ-I(15) in different hospital settings or with other pathological conditions.

## Background

Although a substantial proportion of physical therapy services are delivered each day to inpatients, the literature on satisfaction with inpatient physical therapy is scant. In a previous study [1] we described the translation, cultural adaptation and validation of the Italian version of the Physical Therapy Patient Satisfaction Questionnaire (PTPSQ), developed in 2000 by Goldstein et al. [2]. The original American version of this questionnaire consists of 26 items, of which 20 explore the interaction with the physical therapist and the staff and some environmental factors such as location, cost, and parking.

The introductory section of the PTPSQ helps to clarify that a patient is requested to answer questions specifically relating to his/her physical therapy experience. The specific wording of this introduction is: “You recently received physical therapy services at our facility. Because we strive to deliver the best possible physical therapy services, we are interested in learning from patients how we might improve or enhance our services”. The answer to each item can be chosen from six categories: strongly disagree, disagree, neither agree nor disagree, agree, strongly agree, and no opinion.

The psychometric properties of the original American version were established on a sample of 289 patients from 12 practice settings in the US and indicated high internal consistency (α = 0.99) and good content validity. A one-dimension structure emerged, explaining nearly 83% of the variance, and dominated by satisfaction with the physical therapist interaction. The Italian version of the PTPSQ (PTPSQ-I) was administered to 315 Italian outpatients, and in order to adapt the original questionnaire to a different cultural and social context [3], five items were excluded from the PTPSQ-I, which was renamed the PTPSQ-I(15). The deleted variables were characterized by a very high proportion of missing values, and were related to costs, to physical therapist assistants and to parking availability.

The PTPSQ-I(15) showed good acceptability and high internal consistency (α = 0.905). Divergent validity of the Italian version was investigated with Pearson’s correlations by comparing the PTPSQ-I(15) to the Global Perceived Effect Questionnaire (GPE) [4] and to the Visual Analogue Scale (VAS) for pain [5]. Divergent validity appeared moderate for the GPE (r = −0.33, *P* < 0.001), but not significant for the VAS (r = −0.07, *P* = 0.33). Using factor analysis a 2-factor structure was found, related to perceived “Overall Experience” and “Professional Impression” that together explained 62% of the total variance. A third factor, named “Efficiency and Convenience”, brought explained total variance near to 70%. Both the US version and the Italian version were administered to physical therapy outpatients, who were mostly affected by musculoskeletal dysfunctions. To the authors’ knowledge, the PTPSQ has never been used in a hospital setting to study inpatient satisfaction. As a consequence, we did not know if its psychometric characteristics would be confirmed with respect to a different population and whether the construct would be the same. The aims of this study were: (1) to explore the psychometric properties of PTPSQ-I(15) in a sample of Italian inpatients; and (2) to investigate the relationships between the characteristics of patients and physical therapists and the indicators of satisfaction.

## Methods

This study was conducted on the Physical Medicine and Rehabilitation Unit of a research hospital. All adult (18 years or older) physical therapy inpatients were eligible for the study if they were able to read and speak Italian. Patients who received only a physical therapy evaluation or presented psychiatric or cognitive deficits were excluded. The sample size was based on the “rule of 10” patients per item. Rules-of-thumb vary from four to 10 subjects per variable, with a minimum number of 100 subjects to ensure stability of the variance-covariance matrix [6]. As a consequence, our final expected sample was 150 subjects. The Ethics Committee of the University Hospital S.Orsola-Malpighi of Bologna (Italy) approved the trial (code 32/2011/U/OssN), and all subjects gave their written consent.

Questionnaires were presented by research assistants to each participant, who was assured that his or her physical therapist was blinded to the results. Items were presented to each participant in written form. Participants answered each question verbally, and research assistants filled in the answers. Research assistants could repeat questions but could not change wording. If a participant altered his or her response, the assistant noted the change on the form; if the participant did not choose any answer, the assistant did not mark any box. The levels of patient understanding and the time needed to answer were recorded for each item by the research assistant. Questionnaires were administered before a session of physical therapy treatment, excluding the first session, and in separate rooms, ensuring privacy.

All subjects provided some socio-demographic characteristics and completed the PTPSQ-I(15), the Visual Analogue Scale (VAS) and the 5-point Likert-type scale evaluating the Global Perceived Effect (GPE) of the physical therapy treatment. A sub-sample of randomly selected inpatients took the re-test of PTPSQ-I(15) after the first administration. A simple randomization was employed. After obtaining informed consent and before administering the PTPSQ-I(15), research assistants opened a closed envelope containing the options, “yes” or “no”, indicating whether to include or exclude the patient in the re-test subgroup. Usually the time interval of test-retest reliability studies is chosen from one week to five weeks [7]. We chose a 7-day interim between the first and the second administrations to avoid recall effects on the re-test response (i.e., if the time interval between the test and the retest was too brief) or confounding during the intervening time interval because the time interval between administrations was too long.

### Statistical analysis

Statistical analyses focused on the set of items included in the PTPSQ-I(15) previously determined by adapting the questionnaire originally developed for US outpatients for Italian outpatients. All statistical analyses were performed using PASW Statistics, 18 (Release 18.0.3), SAS (Release 9.2), and R (Release 2.15.2).

#### Acceptability

We recorded the time needed to answer the PTPSQ-I(15), difficulties in comprehension, and missing, changed or multiple responses. On the first day of in-hospital admission, the staff promptly informed patients of the possibility of scheduling follow-up visits at convenient times in order to avoid misunderstandings of Q10 and Q13, and to help them in giving answers appropriately.

#### Reliability

Reliability was investigated with respect to internal consistency and test-retest stability. Test-retest stability aims at evaluating the reliability of a scale by administering the same scale on different occasions and then evaluating the consistency between the observed scores. We re-administered the questionnaire to a sub-sample of patients seven days after the first administration. The Intra-class Correlation Coefficient [ICC(3,1)] [8] was used to test the agreement between the baseline and the 7-day PTPSQ-I(15) total scores and item to item agreement.

#### Internal structure and construct validity

Factor analysis was used to evaluate the internal structure of the scale.

#### Divergent validity

The PTPSQ-I(15) scale was also evaluated by comparing it with the GPE as a measure of the perceived effectiveness of treatment by calculating Pearson and Spearman correlation coefficients. The correlation between the PTPSQ-I(15) and the VAS was also examined to evaluate whether satisfaction as measured by the PTPSQ-I(15) was related to the pain perceived by the patient. In both cases, we expected a negative correlation because the best situation corresponds to the lowest scores on the GPE and the VAS questionnaires.

#### Dependency of satisfaction on external variables

Wilcoxon and Kruskal-Wallis tests were employed to identify the extent to which satisfaction scores were associated with the characteristics of the physical therapist, the facility, and the inpatient.

## Results

### Subjects

A total of 270 patients were admitted to the Physical Medicine and Rehabilitation Unit from April to September 2011, of whom 121 did not meet the inclusion criteria (87 had psychiatric or cognitive deficits, 34 were younger than 18 years). A total of 149 inpatients were eligible, and were asked to participate in the study. Since one of them refused, our sample consisted of 148 inpatients, including 79 females (53.4%) and 69 males (46.6%), whose mean age was 59.47 years (SD = 17.7). Of these, 107 subjects (72.3%) had neurological conditions, and 41 (27.7%) had musculoskeletal disorders. The socio-demographic characteristics of these patients and treatment characteristics are reported in Table 1.

**Table 1:** Characteristics of the sample

**Variable**	**Category**	**n**	**Percentage**
**Gender**	Female	79	53.4%
	Male	69	46.6%
**Age (in classes)**	18-25	5	3.4%
	25-40	22	14.9%
	40-65	63	42.6%
	>65	58	39.2%
**Married**	Yes*	87	58.8%
	No**	61	41.2%
**Working**	Yes	65	43.9%
	No	13	8.8%
	Retired	70	47.3%
**Education**	Elementary	36	24.3%
	Mid school	52	35.1%
	Upper school	39	26.4%
	University	21	14.2%
**Facility recommended by**	Doctor	103	69.6%
	Friends	10	6.8%
	Other Patients	9	6.1%
	Other	26	17.6%
**First treatment in the facility**	Yes	79	53.4%
	No	69	46.6%
**First physical therapy treatment**	Yes	51	34.5%
	No	97	65.5%
**Therapist’s gender**	Female	67	45.3%
	Male	81	54.7%
**Payment**	Direct payment	33	22.3%
	Co-Payment	61	41.2%
	Fully covered (National Health System)	47	31.8%
	Insurance	7	4.7%

* = married, living together. ** = single, widowed, divorced.

### Psychometric characteristics

#### Acceptability

On average, the questionnaire was completed in 4.52 minutes (SD = 1.43 minutes). Only 5 errors (i.e., corrections, deletions, etc.) were noted, corresponding to the 0.22% of the total number of answered questions (15 items for 148 patients, for a total of 2220). No individual item was completely unanswered, no multiple answers were found, and no problems of comprehension of the items were reported.

Table 2 displays the number of missing values for each item, together with the distribution of the responses. Q13 (‘It was easy to schedule visits after my first appointment’) shows the highest number of missing values, which is logical in light of the fact that inpatients generally do not schedule their appointments. The data in Table 2 show that most of the respondents declared high and very high levels of satisfaction (higher than or equal to “4”). Low and very low levels of satisfaction were very rare. Medium levels of satisfaction (“3”) were not particularly frequent, with some exceptions represented by items Q12 (“My first visit for physical therapy was scheduled quickly”), Q15 (“The location of the facility was convenient for me”), and, to a lesser extent, Q09 (“All other staff members were courteous”) and Q19 (“My physical therapist understood my problem or condition”).

**Table 2:** Number of missing values and distribution of responses for each item

**Item**	**Label**	**n**	**% missing**	**Distribution of responses**				
				**1**	**2**	**3**	**4**	**5**
Q07	My privacy was respected during my physical therapy care.	147	0.68%	0.0%	0.0%	6.8%	47.6%	45.6%
Q08	My physical therapist was courteous.	148	0.00%	0.0%	0.0%	7.4%	41.9%	50.7%
Q09	All other staff members were courteous.	147	0.68%	0.0%	0.0%	13.6%	31.3%	55.1%
Q10	The clinic scheduled appointments at convenient times.	132	10.81%	0.0%	0.0%	4.5%	47.0%	48.5%
Q11	I was satisfied with the treatment provided by my physical therapist.	148	0.00%	0.0%	0.0%	8.8%	31.8%	59.5%
Q12	My first visit for physical therapy was scheduled quickly.	136	8.11%	0.0%	0.0%	29.4%	35.3%	35.3%
Q13	It was easy to schedule visits after my first appointment.	117	20.95%	0.0%	0.9%	4.3%	41.0%	53.8%
Q14	I was seen promptly when I arrived for treatment.	146	1.35%	0.7%	0.7%	4.1%	39.7%	54.8%
Q15	The location of the facility was convenient for me	129	12.84%	1.6%	3.9%	20.2%	47.3%	27.1%
Q19	My physical therapist understood my problem or condition.	148	0.00%	0.0%	0.0%	12.2%	34.5%	53.4%
Q20	The instructions my physical therapist gave me were helpful.	146	1.35%	0.0%	0.0%	4.8%	47.9%	47.3%
Q21	I was satisfied with the overall quality of my physical therapy care.	148	0.00%	0.0%	0.0%	4.7%	43.9%	51.4%
Q22	I would recommend this facility to family or friends.	148	0.00%	0.0%	0.0%	2.0%	50.0%	48.0%
Q23	I would return to this facility if I required physical therapy care in the future.	147	0.68%	0.0%	0.0%	2.7%	46.9%	50.3%
Q26	Overall, I was satisfied with my experience with physical therapy.	148	0.00%	0.0%	0.7%	2.7%	50.7%	45.9%

### Reliability

#### Internal consistency

Cronbach’s α for PTPSQ-I(15) was 0.949. In Table 3 we report the α values obtained by deleting one item at a time. Deleting only Q15 (“The location of the facility was convenient for me”) or only Q11 (“I was satisfied with the treatment provided by my physical therapist”) resulted in a slight increase of Cronbach’s α. Otherwise, Q15 (r = 0.261) had a item-total correlation below the critical value of r = 0.30, on the contrary Q11 had a r = 0.526 which is quite good. A modest item-total correlation was also observed for item Q14 (“I was seen promptly when I arrived for treatment”). Therefore, these three items are those which would be left partially unexplained by the PTPSQ-I(15) scale score, obtained by summing the scores observed on all the items.

**Table 3:** Reliability analysis with each question deleted

**Deleted variable**	**Cronbach’s Alpha if Item Deleted**	**Corrected Item-Total Correlation**	**Scale Mean if Item Deleted**	**Scale Variance if Item Deleted**
Q07	0.944	0.829	61.383	49.295
Q08	0.943	0.845	61.365	48.781
Q09	0.944	0.802	61.551	47.231
Q10	0.943	0.877	61.449	48.627
Q11	**0.950**	*0.526*	61.393	51.109
Q12	0.946	0.747	61.692	47.913
Q13	0.944	0.834	61.402	48.526
Q14	0.948	*0.636*	61.308	50.027
Q15	**0.960**	**0.261**	61.766	52.294
Q19	0.944	0.793	61.551	47.608
Q20	0.944	0.830	61.421	48.755
Q21	0.943	0.868	61.402	48.431
Q22	0.946	0.760	61.411	50.037
Q23	0.943	0.867	61.365	49.215
Q26	0.946	0.722	61.449	50.193

Bolded numbers refer to items less correlated with global satisfaction.

#### Internal structure and construct validity

To further analyze the relationships among items and to evaluate whether the PTPSQ-I(15) scale should be parceled into more components, a factor analysis was conducted. Factor analysis was applied to the 15 items included in PTPSQ-I(15) using the principal components extraction method. The results are displayed in Table 4. As is evident from Table 4, two eigenvalues meet the standard rule of an eigenvalue higher than or equal to 1. The first eigenvalue (9.535) is largely the most important, explaining about the 64% of the total variance, whereas the second eigenvalue (1.316) explains only the 8% of the total variance.

**Table 4:** Principal component analysis

**Factor no.**	**Eigenvalue**	**Proportion of variance accounted for**	**Cumulative proportion of variance accounted for**
**1**	**9.535**	0.636	0.636
**2**	**1.316**	0.088	0.723
3	0.971	0.065	0.788
4	0.766	0.051	0.839
5	0.481	0.032	0.871
6	0.406	0.027	0.898
7	0.345	0.023	0.921
8	0.294	0.020	0.941
9	0.237	0.016	0.957
10	0.196	0.013	0.970
11	0.137	0.009	0.979
12	0.102	0.007	0.986
13	0.091	0.006	0.992
14	0.066	0.004	0.996
15	0.058	0.004	1.000

The numbers in bold indicate the factors which met the standard rule of an eigenvalue of 1 or higher.

For the sake of completeness, we proceeded to estimate both a 1-factor and a 2-factor model; results are reported in Table 5. The third column of Table 5 reports the correlations (loadings) between the PTPSQ-I(15) items and the unique factor in the 1-factor model. Although the item regarding the location of the facility (Q15) is not particularly related to this factor, removing this item had no effect on the results of factor analysis. Also, two items, Q14 (“I was seen promptly when I arrived for treatment”) and Q11 (“I was satisfied with the treatment provided by my physical therapist”) demonstrated loadings relatively lower than the others.

**Table 5:** Factor analysis loadings

**Item**	**Description**	**1-Factor**	**2-Factors**	
		**Factor1**	**Factor1**	**Factor2**
**Q09**	All other staff members were courteous.	**0.851**	**0.941**	0.088
**Q19**	My physical therapist understood my problem or condition.	**0.831**	**0.891**	0.132
**Q12**	My first visit for physical therapy was scheduled quickly.	**0.794**	**0.851**	0.126
**Q23**	I would return to this facility if I required physical therapy care in the future.	**0.891**	**0.801**	0.397
**Q08**	My physical therapist was courteous.	**0.875**	**0.770**	0.417
**Q21**	I was satisfied with the overall quality of my physical therapy care.	**0.896**	**0.766**	0.465
**Q10**	The clinic scheduled appointments at convenient times.	**0.905**	**0.754**	*0.502*
**Q07**	My privacy was respected during my physical therapy care.	**0.868**	**0.750**	0.436
**Q20**	The instructions my physical therapist gave me were helpful.	**0.857**	**0.729**	0.451
**Q22**	I would recommend this facility to family or friends.	**0.794**	*0.699*	0.378
**Q13**	It was easy to schedule visits after my first appointment.	**0.860**	*0.668*	*0.559*
**Q26**	Overall, I was satisfied with my experience with physical therapy.	**0.762**	*0.649*	*0.399*
**Q14**	I was seen promptly when I arrived for treatment.	*0.677*	0.251	**0.894**
**Q11**	I was satisfied with the treatment provided by my physical therapist.	*0.573*	0.150	**0.860**
**Q15**	The location of the facility was convenient for me.	0.291	0.132	0.345

Factor analysis loadings: model with 1 factor and with 2 factors respectively (extraction method: Principal Components; rotation method = varimax). The numbers in bold indicate high correlations and in italics indicate moderate correlations.

A 2-factor model was also estimated to verify whether groups of items that were strongly interconnected could be individuated. The two factors were rotated using the varimax criterion in order to improve their interpretation. The loadings are reported in the last columns of Table 5 (where items are arranged to better emphasize their relationships with the extracted factors). We found that the items related to the second factor are those characterized by relatively lower loadings when a unique factor is obtained (third column of Table 5). Some items (in particular, Q13, Q10, Q21, and Q20, but also, to a lesser extent Q07, Q08, Q23, Q22, and Q26) showed moderate loadings with the second factor while being strongly connected to the first. Also, the loadings of these items on the first factor are lower compared to those with the unique factor in the 1-factor model. This suggests a less than clear distinction between groups in a 2-factor model.

#### Test-retest stability

There were 73 subjects who re-took the PTPSQ-I(15), and almost all of them (72 out of 73) had complete data. At the time of re-administration, physical therapy treatment was still in progress. The correlation between the two totals was highly significant (ICC = 0.996, 95% CI: 0.994-0.998). We also analyzed the consistency between the scores assigned to each item in the two administrations of the questionnaire. Data in Table 6 show the high consistency for all variables: for some items the ICC(3,1) is equal to 1, signaling perfect consistency between the answers given on the two occasions.

**Table 6:** Test-retest repeatibility

**Item**	**n**	**ICC(3,1)**	**95% Confidence limit**	
**Q07**	72	1		
**Q08**	72	1		
**Q09**	72	1		
**Q10**	72	1		
**Q11**	72	.986	.978	.992
**Q12**	72	.972	.956	.983
**Q13**	72	.972	.956	.983
**Q14**	72	.988	.981	.993
**Q15**	72	1		
**Q19**	72	.984	.974	.990
**Q20**	72	.976	.962	.985
**Q21**	72	1		
**Q22**	72	1		
**Q23**	72	1		
**Q26**	72	1		
**TOTAL**	72	.996	.994	.998

Test-retest repeatability (day 1 and day 7) was calculated using the ICC(3,1) index.

#### Divergent validity

The divergent validity was measured by calculating the Pearson and Spearman correlation coefficients between the total score of the PTPSQ-I(15) and scores on the VAS and GPE. The PTPSQ-I(15) total showed a modest and significant negative correlation with GPE, (r = −0.502, *p* < 0.0001; S = −0.516, *p* < 0.0001). The correlation with VAS was low and not significant (r = −0.17, *p* = 0.07; S = −0.113, *p =* 0.248).

Following Goldstein and co-investigators [2], attention was also focused on a set of items which can be considered as the best indicators of overall satisfaction: Q22 (‘I would recommend this facility to family and friends’), Q23 (‘I would return to this facility in the future’), and Q26 (‘Overall satisfaction with the physical therapy experience’). A new total was obtained by summing the scores on the remaining 12 items, and the relation between this reduced total and the three mentioned items was evaluated using again the Pearson correlation coefficient. The obtained correlations were all high and significant (correlations: with Q22: r = 0.73 and S = 0.70; with Q23: r = 0.86 and S = 0.83; with Q26: r = 70 and S = 0.68; *p* values were lower than 0.0001 in all cases), indicating a fair correlation between the total and each criterion variable.

#### Dependency of satisfaction on external variables

We then analyzed the relationship of satisfaction, as measured by the PTPSQ I(15) total score, to the characteristics of the patients, of the facility where they received the therapy, and of the therapists. The explanatory factors were all categorical. Analysis of Variance (ANOVA) was used to test whether the means of the total score significantly differed according to the levels of each possible explanatory variable. The distribution of total scores turned out to be not normal. We therefore used a non-parametric ANOVA approach, i.e., the Kruskal-Wallis test. The Wilcoxon test was used for explanatory variables having only two levels. Instead of relying upon the actual values of the response (in our case, the total score), these tests are based on the response’s *ranked* values. Thus, the values taken by the response are ordered from the smallest to the largest, and each value is assigned to its rank, i.e., its position in the ordered sequence of values. The means of the ranks for subjects are grouped according to the levels of the considered (categorical) explanatory variable, and the differences among the means of ranks are tested.

For each explanatory variable, Table 7 displays the means (and the means ranks) of the total score within the groups by each level of the variable and the *p*-values for the null hypotheses that the mean ranks are all equal. A low *p*-value indicates that the null hypothesis has to be rejected in favor of the alternative hypothesis that at least two groups have mean ranks different one from another. Groups with a too few cases were not included in the analysis. Specifically, the group of patients aged 18–25 with only 4 valid cases and the group of patients covered by insurance with only 5 valid cases were excluded.

**Table 7:** Relationship between satisfaction and Patients Characteristics

**Variable**	**Levels**	**n**	**Mean**	**Mean of ranks**	**P-value**^**(1)**^
**Gender**	Female	59	**67.05**	**58.57**	*0.0898*
	Male	48	64.38	48.39	
**Age (in classes)**	18-25	4	73.50	n.c.^(2)^	0.5285
	25-40	19	66.89	58.74	
	40-65	47	64.94	51.29	
	>65	37	65.65	49.45	
**Married**^**(3)**^	Yes	62	65.89	53.86	0.9570
	No	45	65.80	54.19	
**Education**	Elementary	23	66.83	57.70	0.5924
	Mid school	39	64.59	48.63	
	Upper school	29	66.00	56.14	
	University	16	67.25	57.91	
**Working**	Yes	54	66.28	57.02	0.5876
	No	12	64.42	52.08	
	Retired	41	65.71	50.59	
**Facility recommended by**	Doctor	69	64.16	*45.93*	**0.0028**
	Friends	10	67.00	*61.65*	
	Other	20	69.20	**69.63**	
	Other Patients	8	70.63	**74.94**	
**First treatment in the facility**	Yes	56	66.32	57.04	0.2852
	No	51	65.33	50.66	
**First Physical Therapy treatment**	Yes	35	63.69	46.24	*0.0700*
	No	72	66.90	**57.77**	
**Therapist’s gender**	Female	41	64.39	45.95	***0.0336***
	Male	66	66.76	**59.00**	
**Combination of gender patient/PT**	Male patient, female PT	18	62.33	*38.28*	***0.0528***
	Both Females	23	66.00	*51.96*	
	Both Males	30	65.60	*54.45*	
	Female patient, male PT	36	67.72	**62.79**	
**Payment**	Co-Payment	45	61.62	35.04	**<0.0001**
	Direct payment	33	68.09	**63.02**	
	Fully covered	24	69.58	**66.52**	
	Insurance	5	71.20	n.c.^(3)^	

Means of the total within groups of patients, and results of tests on the equality of the means (Kruskal-Wallis or Wilcoxon test) are showed.

^(1)^*p* value: the null hypothesis is that the means of the ranks are all equal one to another; the alternative hypothesis is that at least two means (of ranks) differ.

^(2)^ For each explanatory variable, in the case of rejection of the null hypothesis of equal means (of ranks) levels with statistically different means are specified. Means with different format (normal, bold, italics, or underlined) are statistically different one from another (at the level α=0.05). Instead, means having at least one common format are *not* statistically different.

^(3)^ The group of patients was not considered due the too low number of cases.

Yes = married, living together; No = single, widowed, divorced.

With explanatory variables with only two levels, rejecting the null hypothesis is equivalent to conclude that the mean ranks (in the two groups) are statistically different. For explanatory variables with more than two levels, multiple comparisons are needed to determine which groups have significantly different means. To assure an adequate post-hoc control of the Type I error, the non-parametric procedure illustrated by Siegel and Castellan [9] (available in the package “pgirmess” in R) was used.

With respect to the ‘technical’ aspects (attended facility, method of payment, the way the patient knew about the facility, etc.), significant relationships were found between the total and the method of payment (*p* < 0.0001). We observed that the most satisfied patients are those who do not pay for the therapy, while the least satisfied are those who partially pay for it. Also, patients who received their therapy for the first time were less satisfied than other respondents. Further, the level of satisfaction for patients who had been referred to the facility by their doctor was significantly lower compared to that of patients who were recommended by former patients or who responded “Other” and selected the facility using sources other than the pre-specified ones, most probably using the Internet, or guided by the reputation of the selected center.

Regarding the impact of the gender of the physical therapist on the level of satisfaction, we observed that although females were significantly more satisfied than males as a group, we also found that patients treated by male physical therapists were more satisfied on average. Also, the least satisfied patients in any subgroup were males treated by female therapists. The relationships between the considered totals and other socio-demographic characteristics (education level, age class, marital status) of the patients were not particularly relevant.

## Discussion

Inpatient satisfaction with physical therapy has rarely been reported. The reasons for this may be that the severity of patients is so high as to make it difficult to administer a questionnaire, brief lengths of stay in many instances, the challenges that patients might not be able to accurately identify who among the professionals they see are physical therapists, or also which treatments they received were physical therapy interventions.

In Italian Physical Medicine and Rehabilitation Units, inpatients can easily identify which type of professional they see, due to the different uniforms or dress worn by a particular group and/or the identification badges, showing the name, the picture and the qualification of each person in contact with patient. Moreover, somewhat differently than the US, physical therapy in Italy is delivered only by physical therapists because other kinds of personnel such as the “physical therapists assistants” do not exist. In this paper, we described a validity study of the Italian version of the PTPSQ [PTPSQ-I(15)] in a sample of inpatients receiving physical therapy treatments in the Physical Medicine and Rehabilitation Unit of a research hospital. The PTPSQ-I(15) administered to inpatients demonstrated good acceptability, evidenced by the short time required for completion and the good comprehension of the items. Internal consistency was strong even if it did not match the findings of the original US version.

Comparatively, we found lower overall values for internal consistency (Cronbach’s α) than in our previous studies on the same questionnaire (0.905) administered to outpatients [1] and with to respect to data on the Italian version of the Physical Therapy Outpatient Satisfaction Survey (0.758 for Enhancers, 0.847 for Detractors, 0.885 for Location, and 0.706 for Cost) [10]. Test-retest stability at seven days of the PTPSQ-I(15) was also strong and almost perfect, but we cannot compare this result with any other investigation on this questionnaire due to the absence of previous similar analysis. The previously cited Italian version of the Physical Therapy Outpatient Satisfaction Survey demonstrated lower test-retest stability (Intra-class Correlation Coefficients 0.769 for Enhancers, 0.893 for Detractors, 0.862 for Location, and 0.862 for Cost) [10].

Divergent validity was moderate for the GPE and strong for the VAS. Our results are in line with the findings generated by our previous study on the same questionnaire [1] and by the findings by Kelly [11], George and Hirsh [12], Skolasky et al. [13], and Vanti et al. [10]. The conclusion of the systematic review of Hush et al. [14] appears corroborated, i.e., a weak relationship exists between clinical outcome and satisfaction. However, it must be noted that other investigators [15,16] have found a significant inverse relationship between pain associated with different health conditions and satisfaction with care in different settings at short-term follow-ups.

Our factorial analysis showed a one-factor structure with a strong interconnection among items that is markedly similar to the findings obtained by Goldstein and colleagues [2].

Satisfaction with physical therapy in Italian inpatients appears as “compact” concept. This is consistent with the study of Mangset et al. [17], who found a main core factor contributing to patient satisfaction, specifically “to be treated with respect and dignity”. Also Medina-Mirapeix et al. [18] observed that management and relational continuity explained most of the variance in patient satisfaction.

Italian inpatients receive their treatment in a structured setting where they are in contact with a variety of health care professionals, including various technical and assistive personnel. Satisfaction with physical therapy in this kind of treatment setting may be a concept comprising technical, relational, and logistic aspects of care.

It is interesting that the most satisfied Italian patients are those who do not pay for therapy, while the least satisfied are those who partially pay for it. This result is different from that found by Issa et al., who showed that co-pays did not affect patient satisfaction with post-operative physical therapy following total hip arthroplasty [19]. In our opinion, this opposite finding may be due to cultural attitudes specific to Italians, because we found the same result also in our previous study on outpatients [1].

Interestingly, we found opposite results in Italian inpatients compared to outpatients about the relationship between satisfaction and admission mode or referral (doctor, friend, former patient), possibly because the patient’s expectations for treatment in hospital setting are different compared to those of outpatients. These results are similar to those found by Murante et al. [20], who showed that living in the hospital area (and, more probably, referred by a doctor) negatively affected patients’ overall experience. In our study the level of satisfaction was higher for those individuals who were recommended by former patients or who answered “Other”, who appear to have made an informed decision specifically on the basis of their needs and preferences. Our results also demonstrated that being hospitalized more than once negatively affects overall experience, consistent with the findings of another study of an Italian inpatient population [20].

As for the impact of the gender of the physical therapist on the level of satisfaction, we found that inpatients treated by male physical therapists were more satisfied on the average, whereas our previous study on outpatients and other peer-reviewed literature showed the opposite [1,10,21-23]. The reasons for these results are difficult to interpret, unless we hypothesize that Italian inpatients somehow inject their own gender biases in the situation so that they attribute greater intelligence, aptitude for science, strength or safety from male professionals without reason. Intriguingly in a psychiatric practice, a correlation between gender preference and sex-role stereotypes of patients has been demonstrated [24]. Another reason could be simply related to the different characteristics of the physical therapists actually involved in our studies.

The relationship between the socio-demographic characteristics of the patients and their satisfaction appeared to have little consequence. This result confirms the findings from other studies which showed that age, gender, ethnicity, and marital and socioeconomic status are weak predictors of patient satisfaction [17,25], but is different from our previous findings using an outpatient sample [1,10]. Older and less educated outpatients appeared, in fact, more satisfied, according to another study on Italian inpatients [20]. This difference could be due to the specific characteristics of the hospital involved in our research (i.e., a Physical Medicine and Rehabilitation Unit).

The main limitations of this study concern sample size and the generalizability of findings from only one Physical Medicine and Rehabilitation Unit. As a consequence, we do not know if these results would be replicated in other Italian rehabilitation hospitals or would be sustained across different health conditions. Another limit is related to the approach used in determining psychometric properties. We chose the Classical Test Theory, but we are aware that there are other modern approaches based on Item Response Theory, such as Rasch Analysis, that are now available to evaluate questionnaire psychometrics.

## Conclusions

The overall strong psychometric properties of PTPSQ-I(15) allow us to recommend the use of this questionnaire to evaluate satisfaction. However, we must caution therapists that the construct referred to as “patient satisfaction” may be setting dependent, even when the same instrument is used. Further studies are suggested on the concurrent validity and on the psychometric properties of the PTPSQ-I(15) in different hospital settings or pathological conditions. Moreover, the construct of “patient satisfaction” should be explicated by comparing similarities and differences between outpatients and inpatients receiving physical therapy.

## Abbreviations

CI: Confidence Interval; GPE: Global Perceived Effect; ICC: Intra-class Correlation Coefficient; PTPSQ: Physical Therapy Patient Satisfaction Questionnaire; SD: Standard Deviation; US: United States of America; VAS: Visual Analogue Scale.

## Competing interests

The authors declare that they have no competing interests.

## Authors’ contribution

CV and PP designed the study. FB, DC and MM were responsible for data collection. RP was responsible for data analysis, together with AG, CV, FSV and PP contributed to interpretation of data. CV, PP, RP and AG drafted the manuscript, together with MM and FSV. All authors critically revised the manuscript. All authors read and approved the final manuscript.

## Pre-publication history

The pre-publication history for this paper can be accessed here:

http://www.biomedcentral.com/1471-2474/15/135/prepub
